# Sugarcane Nitrogen Concentration and Irrigation Level Prediction Based on UAV Multispectral Imagery

**DOI:** 10.3390/s22072711

**Published:** 2022-04-01

**Authors:** Xiuhua Li, Yuxuan Ba, Muqing Zhang, Mengling Nong, Ce Yang, Shimin Zhang

**Affiliations:** 1 Guangxi Key Laboratory of Sugarcane Biology, Guangxi University, Nanning 530004, China; lixh@gxu.edu.cn (X.L.); mqzhang@ufl.edu (M.Z.); 2 School of Electrical Engineering, Guangxi University, Nanning 530004, China; 1812302001@alu.gxu.edu.cn; 3 Beijing Institute of Remote Sensing Equipment, Beijing 100854, China; 4 IRREC-IFAS, University of Florida, Fort Pierce, FL 34945, USA; 5 School of Agriculture, Guangxi University, Nanning 530004, China; 20040047@gxu.edu.cn; 6 Department of Bioproducts and Biosystems Engineering, University of Minnesota, Saint Paul, MN 55108, USA; ceyang@umn.edu

**Keywords:** sugarcane, multispectral image, canopy nitrogen concentration, irrigation classification, UAV

## Abstract

Sugarcane is the main industrial crop for sugar production, and its growth status is closely related to fertilizer, water, and light input. Unmanned aerial vehicle (UAV)-based multispectral imagery is widely used for high-throughput phenotyping, since it can rapidly predict crop vigor at field scale. This study focused on the potential of drone multispectral images in predicting canopy nitrogen concentration (CNC) and irrigation levels for sugarcane. An experiment was carried out in a sugarcane field with three irrigation levels and five fertilizer levels. Multispectral images at an altitude of 40 m were acquired during the elongating stage. Partial least square (PLS), backpropagation neural network (BPNN), and extreme learning machine (ELM) were adopted to establish CNC prediction models based on various combinations of band reflectance and vegetation indices. The simple ratio pigment index (SRPI), normalized pigment chlorophyll index (NPCI), and normalized green-blue difference index (NGBDI) were selected as model inputs due to their higher grey relational degree with the CNC and lower correlation between one another. The PLS model based on the five-band reflectance and the three vegetation indices achieved the best accuracy (*R*_v_ = 0.79, *RMSE*_v_ = 0.11). Support vector machine (SVM) and BPNN were then used to classify the irrigation levels based on five spectral features which had high correlations with irrigation levels. SVM reached a higher accuracy of 80.6%. The results of this study demonstrated that high resolution multispectral images could provide effective information for CNC prediction and water irrigation level recognition for sugarcane crop.

## 1. Introduction

As the most important sugar crop, sugarcane is mainly grown in tropical and subtropical areas and provides approximately 80% of the world’s sugar [1,2]. The growth of sugarcane is closely related to fertilizer, water, and radiation intensity. Evaluating the growth situation of sugarcane in a timely manner, and adjusting the field management strategy accordingly, is of great significance to the yield and quality of sugarcane. In recent years, remote sensing with spectral images at different scales has been considered an effective high-throughput phenotyping solution for predicting the growth and yield of crops.

Large-scale spectral imagery can cover an area ranging from 25 to 3600 km^2^ per image. Spatial resolutions generally range from more than 1 m to tens of meters, and some data can reach 0.3 m [3,4]. They are mainly obtained by satellites, such as Sentinel [5], Gaofen (GF) [6], Landsat [7,8], GeoEye [9], and QuickBird [10], which are normally provided by governments or commercial companies. Their main agricultural applications include land cover and land use investigation, vegetation classification, and crop yield forecasting. However, due to the limitations of low spatial resolution and fixed revisit cycles, it has formidable deficiencies for small-scale applications, which usually need more subtle and frequent data acquisition for crop growth monitoring [11].

Middle-scale spectral imagery can provide data with submeter spatial resolutions ranging from less than 1 m to a few meters [4,12]. These kinds of images are mainly acquired by aviation aircraft platforms, integrated with multispectral or hyperspectral imaging sensors, at an altitude of several kilometers with fairly large coverage and high spatial resolutions [13,14]. However, these kinds of platforms are not popular due to their high costs.

Small-scale spectral imagery is usually acquired by unmanned aerial vehicles (UAVs) and can normally cover up to hundreds of hectares [3,4]. With the rapid development of UAVs, combined with the increasing availability and decreasing cost of spectral imaging sensors, opportunities to capture spectral images with high spatial and spectral resolutions have abounded. UAV-based remote sensing systems can easily reach spatial resolution of centimeter, which means that they are more sensitive to spatially heterogeneous information. Over the past 10 years, UAVs, especially drones, have been rapidly accepted and popularized to acquire reliable field crop information, weather permitting [15]. They can provide subtle information about the crop canopies in every inch of a field, which is difficult to acquire via ground-based scouting by people, especially for tall plants. As such, these systems save labor and time [16,17].

Many previous studies have shown that crop yield [18,19], nitrogen (N) status [20,21,22], protein content [23,24] and water stress [25,26] can be predicted by drone-based multispectral and RGB imagery. When establishing models for different crops, various spectral features including spectral reflectance, existed vegetation indices (VIs), and newly proposed VIs can be used as input variables. Taking N prediction as an example, Peng et al. used the ratio vegetation index (RVI), the normalized difference red-edge index (NDRE) and the terrestrial chlorophyll index (TCI), to predict potato N status [27]. Zhang et al. used RVI and the normalized difference vegetation index (NDVI) to predict rice N status [28]. Osco et al. used NDVI, NDRE, the green normalized difference vegetation (GNDVI), and the soil-adjusted vegetation index (SAVI) to predict maize leaf nitrogen concentration (LNC) [29]. For water status prediction, SAVI [25], the normalized green-red difference index (NGRDI) [26], NDVI [30], NDRE [31] and so on, were reported in different studies for different crops. One of the main reasons why different spectral features are used in different crops is that the physiological characteristics and canopy distribution characteristics of different crops are different. Sugarcane is a tall and dense sugar crop. Unlike other crops, its stalk is the main raw material for sugar production, and it is an important organ for accumulating nutrients. Sugarcane has a long growing season, blooms late, and most of the time its canopy contains only leaves. Therefore, it is of practical significance to find suitable spectral features and establish corresponding growth prediction models for sugarcane.

Preliminary studies of remote sensing for sugarcane have also been conducted in recent years. Sugarcane planting areas classification [32], and large-scale yield prediction, [33,34] were reported based on satellite images. Predictions of sugarcane canopy nitrogen concentration (CNC) or LNC based on hyperspectral data [35] or hyperspectral imagery [36] were also reported. However, studies on CNC prediction and irrigation level classification based on high resolution multispectral imagery were seldomly reported.

In terms of modeling algorithms, both traditional machine learning algorithms and newly developed deep learning algorithms were used. Each has its own advantages and disadvantages. Deep learning algorithms have better performance in the case of sufficient samples. Ma et al. developed a county-level corn yield prediction model based on the Bayesian Neural Network (BNN) using multiple publicly available data sources over 20 years, including satellite images, climate observations, soil property maps and historical yield records [37]. Khaki et al. proposed a convolutional neural network model called YieldNet to predict corn and soybean yield based on MODIS products [38]. Yang et al. tried to use one-year hyperspectral imagery to train a CNN classification model to estimate corn grain yield [39]. Prodhan et al. monitored drought over South Asia using a deep learning approach with 16 years of remote sensing data [40]. It can be seen that a large volume of image data, as well as ground truth data in years, were commonly needed to provide a sufficient dataset to train a deep learning network. To collect this large number of data samples is very challenging. Therefore, the dataset of deep learning is difficult to produce in some circumstances. By contrast, the traditional machine learning methods which are generally based on statistics are suitable for most of the modeling problems when relatively small number of samples are available [41,42]. Partial least squares (PLS), extreme learning machines (ELMs), backpropagation neural networks (BPNNs), support vector machine (SVM), and others, have been widely used in crop nutrient predictions. For example, Li et al. [43] used PLS to establish 12 models of fruits and seeds for rapid analysis and quality assessment. Kira et al. established a model for estimating the chlorophyll and carotenoid contents of three tree varieties based on BPNN [44]. Chen et al. constructed a BPNN model to invert rice pigment content with several spectral parameters as input [45]. Pal et al. used an ELM algorithm to classify land covers with multispectral and hyperspectral data [46]; it achieved a better classification accuracy than models established with BPNN and support vector machine (SVM), with far less computational complexity. Different machine learning methods can suit for different cases depending on variable quantity, sample quantity, and the potential relationship between inputs and output.

In this study, in order to monitor the growth status of sugarcane canopies by a high throughput method, high-resolution multispectral images of an experimental sugarcane field were obtained by a low-altitude UAV. The objectives of this study were (1) to determine the sensitive spectral features for the predictions of the CNC and irrigation levels; (2) to establish the prediction models of the CNC based on different machine learning algorithms such as PLS, BPNN, and ELM; (3) to establish classification methods of irrigation levels based on SVM and BPNN.

## 2. Materials and Methods

### 2.1. Study Area

**Figure 1:**
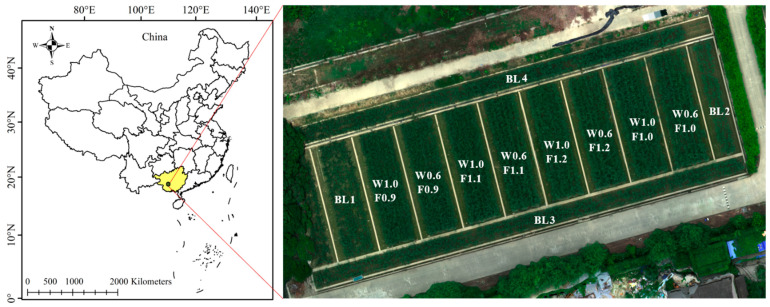
The study site and the field management layout with different irrigation and fertilization levels based on the captured multispectral image (displayed in RGB). W0.6 represents the irrigation rate of 180 m^3^/ha, W1.0 represents the irrigation of 300 m^3^/ha; F1.0 represents the standard fertilization rate, F0.9 represents 90% of the amount of F1.0, F1.1 represents 110% of F1.0 and F1.2 represents 120% of F1.0; BL1, BL2, BL3, BL4 indicate that four blank plots without fertilizer and irrigation.

The sugarcane experimental field was in Nanning, Guangxi Autonomous Region, China (latitude 22.84° N, longitude 108.33° E), as shown in Figure 1. From the captured multispectral image (displayed in RGB) in the right of Figure 1, it can be seen that the experimental field had 12 plots with concrete partitions. Three irrigation treatments and five fertilization treatments were applied in the field. Urea, calcium magnesium phosphate, and potassium chloride were chosen as N, phosphorus (P), and potassium (K) fertilizers, respectively. Eight plots with different irrigations and fertilizers and four blank plots without fertilizer and irrigation (denoted by BL) were set in the field. Concrete partitions at a depth of 1.2 m were built between each plot to prevent water and fertilizer infiltration. The planted seedlings were limited to 975,000 plants per hectare. The two irrigation treatments included 180 m^3^/ha (denoted by W0.6) and 300 m^3^/ha (denoted by W1.0), while the four fertilizer treatments included F1.0 (250 kg/ha of N, 150 kg/ha of P_2_O_5_, 200 kg/ha of K_2_O), F0.9 (90% of the amount of F1.0), F1.1 (110% of F1.0) and F1.2 (120% of F1.0). Water and fertilizer were applied via drip irrigation pipes. Micronutrient fertilizers were equally applied to all the plots except the blank plots. The eight plots had the same size of 20 m × 6 m, with their different treatments denoted by W0.6F0.9, W0.6F1.0, W0.6F1.1, W0.6F1.2, W1.0F0.9, W1.0F1.0, W1.0F1.1 and W1.0F1.2.

The seed canes were planted on 24 March 2018. The seedling fertilizers, which accounted for 30% of the total fertilizer application, were applied on 11 May (28 days after planting). The tillering fertilizers, which accounted for 70% of the total fertilizer application, were applied on 29 June (67 days after planting). The irrigation schedule is listed in Table 1.

**Figure 2:**
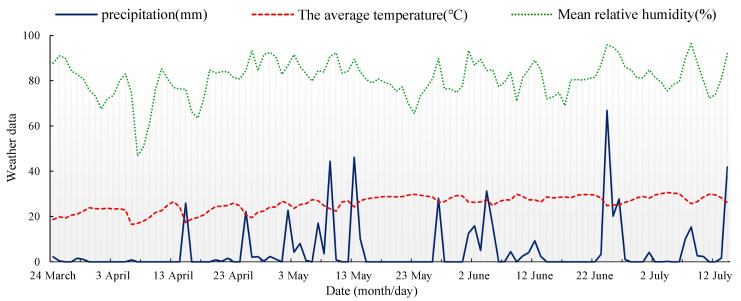
Meteorological data of the experimental field.

Rainfall was another way that water entered the open field. The rainfall in this field was 509.8 mm from the day of planting (24 March) to the day of image acquisition (11 July), and the monthly average rainfall was 127 mm. The meteorological conditions of the experiment field, including precipitation (without the irrigation), temperature and mean relative humidity, are shown in Figure 2. It can be seen that there was almost no rainfall for 15 days before the day of image acquisition. As such, the last event of water input in a large amount was the controlled irrigation on 4 July, which was a week before canopy image acquisition. This means that the rainfall had a very limited influence on the remote evaluation of water stress conditions under specific irrigation amounts.

### 2.2. Data Collection

**Figure 3:**
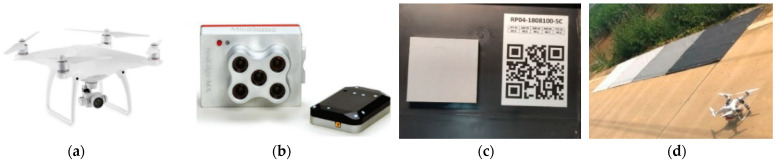
The image acquisition system. (**a**) DJI Phantom 4 Pro; (**b**) RedEdge-MX multispectral image sensor; (**c**) reflectance correction panel of RedEdge-MX; and (**d**) calibration tarps.

The multispectral images were captured at noon on 11 July 2018 (109 days after planting), in the elongating stage. The weather was sunny, cloudless, and windless. The image acquisition system was mainly composed of a drone modeled Phantom 4 Pro (DJI, Shenzhen, China) and a multispectral image sensor RedEdge-MX (MicaSense, Seattle, WA, USA), as shown in Figure 3a,b, respectively. RedEdge-MX image sensor has five spectral bands at 475 nm (blue, B), 560 nm (green, G), 668 nm (red, R), 717 nm (red edge, RE), and 840 nm (near infrared, NIR), and is equipped with a light intensity sensor and a reflectance correction panel (Group VIII, USA, Figure 3c) for radiation correction. The optical intensity sensor can correct the influence caused by changes in sunlight on the spectral images during a flight, and the fixed reflectance correction panel can be used for reflectance transformation. The drone flew at an altitude of 40 m, with 85% forward overlap and 85% side overlap. The time interval of image acquisition was 2 s, and the ground sample distance (GSD) was 2.667 cm. Four calibration tarps with reflectivity of 5%, 20%, 40% and 60%, respectively, were also placed at the open space next to the field before image acquisition, as shown in Figure 3d. Two hundred and sixty multispectral images were finally collected.

### 2.3. Ground Sampling and CNC Determination

Each plot was divided into three sampling areas. Each sampling area was divided into nine grids, and one plant was randomly selected to collect the first fully unfolded leaf for each grid. Nine leaves were collected to form a leaf sample for each sampling area. A total of 36 samples were finally collected, and these were immediately brought back to the laboratory for N determination. All the samples were oven-dried at 105 °C for 30 min and afterward at 75 °C for about 24 h until at a constant weight. The dried leaves were ground and weighed to 0.3 g, and the Kjeldahl method [47] was used to determine the total nitrogen (*TN*, %) content. The *TN* of those first-leaf samples, which were then considered as the CNC (%), could be calculated by Equation (1).

(1) $$TN\;\%=\frac{\left(V_{1}-V_{0}\right)\times C\times 0.014}{m}\times 100\%$$

where % represents the unit of *TN* and CNC; *V*_1_ is the consumption volume of the acid standard solution, mL; *V*_0_ is the titration blank volume, mL; C is the concentration of the acid standard solution, mol/L; 0.014 is the 1 mol standard titration solution equivalent to the weight of N, g; *m* is the weight of the sample, g.

### 2.4. Multispectral Image Preprocessing

**Figure 4:**
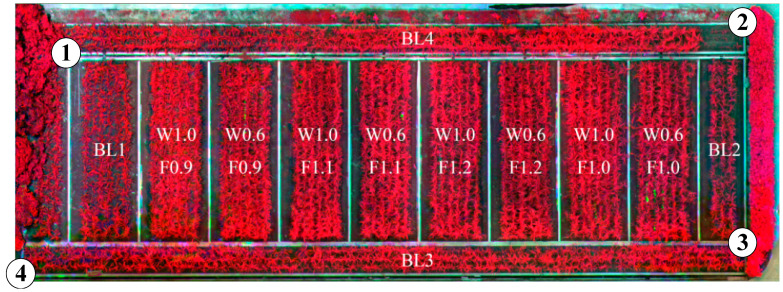
The false-color (NIR, R, and G) mosaic image of the sugarcane experimental field. The white-circled numbers represent the four ground control points.

Pix4DMapper software (Pix4D, Prilly, Switzerland) was used to generate the mosaic image from the 260 original multispectral images, as shown in Figure 4. The mosaic image was then imported and processed in ENVI software (L3Harris Technologies, Melbourne, FL, USA). Two preprocessing steps were conducted in ENVI, including radiation correction and geometric correction. 

Radiation calibration was implemented using the radiometric correction module in ENVI. The “empirical line” method was selected, since four calibration tarps with known reflectivity were captured in the image. An empirical line was fitted by comparing the DN values and the reflectivity of the tarps. Subsequently, all the DN values in the mosaic image were able to be converted into reflectivity. 

Geometric correction was conducted to eliminate the distortion. Four ground control points were selected at four corners of the field, as marked in the false-color image in Figure 4. The “image to map” function was selected to implement geometric correction with the coordinate information of the ground control points. “Nearest neighbor”, which avoids introducing new pixel values, was used to resample the image to the same coordinate system (UTM projection, WGS-84 datum) as that of the ground control points. 

**Figure 5:**
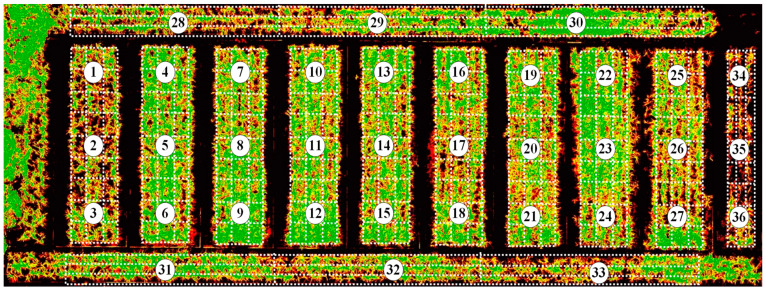
NDVI image of the extracted canopy. The 36 white circled numbers represent 36 sampling areas, each sampling area was further divided into nine grids as shown by the white dashed lines.

In order to extract the region of interest (ROI) out of the background, a classification method, decision tree (DT), was used to extract the sugarcane canopy from the soil, weeds, shadow, concrete, and other interfering background features. Figure 5 shows the NDVI image of the extracted canopy, and the white dots in the figure represented 36 sampling areas. To enhance sample quantity, each area was further divided into nine grids, which were approximately 1.5 m × 2.0 m in size. The average value of each grid was calculated as the spectral sample. Therefore, a total of 324 spectral samples were extracted. 

### 2.5. Feature Extraction and Data Analysis Methods

#### 2.5.1. Extraction of VIs

VIs have been widely used to qualitatively and quantitatively evaluate vegetation cover varieties and crop vigor. NDVI is the most commonly used VI, and it is also one of the important parameters closely related to crop chlorophyll and N concentration. Besides NDVI, nine other commonly used VIs (as shown in Table 2) were also selected to compare their effects on predicting the CNC. The optimal VI or a combination of VIs was used to build the prediction models of the CNC and the irrigation levels.

#### 2.5.2. Grey Relational Analysis

Grey relational analysis (GRA), also called grey incidence analysis (GIA), is an important part of grey system theory, which was developed by Julong Deng [57]. At its core, it works to determine the primary and secondary relationships between various factors by calculating grey relational degree (*GRD*). The higher the *GRD* value of any two factors, the more consistent the change between those two factors. Therefore, it can be used to select the factor with the greatest influence [58]. Let the reference sequence be $X_{0}=\left\{x_{0}\left(k\right),k=1,2,\cdots ,n\right\}$ and the comparison sequence be $X_{i}=\left\{x_{i}\left(k\right),k=1,2,\cdots ,n\right\}$. The *GRD* value between *X*_0_ and *X_i_* is calculated by Equations (2) and (3).

(2) $$GRD=\frac{1}{n}\sum_{k=1}^{n}\gamma \left(x_{0}\left(k\right),x_{i}\left(k\right)\right)$$

(3) $$\gamma \left(x_{0}\left(k\right),x_{i}\left(k\right)\right)=\frac{\underset{i}{\min}\underset{k}{\min}\left|x_{0}\left(k\right)-x_{i}\left(k\right)\right|+\rho \underset{i}{\min}\underset{k}{\min}\left|x_{0}\left(k\right)-x_{i}\left(k\right)\right|}{\left|x_{0}\left(k\right)-x_{i}\left(k\right)\right|+\rho \underset{i}{\min}\underset{k}{\min}\left|x_{0}\left(k\right)-x_{i}\left(k\right)\right|}$$

where ρ is the identification coefficient, and its value range is 0–1, taken here as 0.5. 

The GRA was conducted between all the spectral features and the CNC, which were all normalized. The *GRD*, which was higher than 0.8, reflected that the VI had a very strong influence on the CNC.

#### 2.5.3. Correlation Analysis

Correlation coefficient (*R*) [59] can reflect the degree of the linear correlation between two datasets. It can be calculated by Equation (4).

(4) $$R=\frac{\sum_{i=1}^{n}\left(x_{i}-\bar{x}\right)\left(y_{i}-\bar{y}\right)}{\sqrt{\sum_{i=1}^{n}{\left(x_{i}-\bar{x}\right)}^{2}}\sqrt{\sum_{i=1}^{n}{\left(y_{i}-\bar{y}\right)}^{2}}}$$

where *n* is sample size, $x_{i}$ and $y_{i}$ are the individual sample points indexed with *i*; $\bar{x}$ and $\bar{y}$ are the means of $x_{i}$ and $y_{i}$ for *n* samples.

The higher the absolute value of *R*, the higher the linear correlation between the two factors. It is generally considered that 0.7 ≤ |*R*| < 1 indicates a very high correlation, when 0.4 ≤ |*R*| < 0.7, it indicates a significant correlation, and when |*R*| < 0.4, it indicates a low correlation. Correlation analysis can be applied for multiple purposes in modeling, including: (1) to analyze the correlations between input variables and predictors to determine sensitive variables; (2) to analyze the correlations between multiple variables, during which only the variables with significant correlations should be utilized in order to simplify the complexity of the model; (3) to analyze the correlation between the predicted values of a model and the measured values, and to evaluate the effect of the model.

In this study, the correlations between the spectral features were analyzed to pick proper variables with less redundant information for CNC modeling and irrigation level classification.

### 2.6. Modeling Algorithms

At present, there are many machine learning algorithms. Based on previous researches, four algorithms were selected after comprehensive consideration, as shown in Table 3. 

PLS, BPNN and ELM were selected for CNC modeling, and the simple validation method, hold-out [60], was selected for model validation. All 324 samples were divided into calibration set and validation set according to the ratio of 7:3. 

SVM and BPNN were selected for irrigation level classification. Three-fold cross validation [61] was used to produce more validation samples, and, therefore, to generate a comprehensive confusion matrix of the classification results. 

The PLS algorithm builds a model by minimizing the sum of the squares of the errors. It combines the advantages of multiple linear regression, canonical correlation analysis and principal component analysis. BPNN has the characteristics of self-learning and self-adaptation, showing a strong ability to fit nonlinear functions; it also has a strong anti-interference ability and may be suitable for complex field environments. The ELM algorithm allows for the random generation of the weights and thresholds between the input layer and hidden layers; users only need to denote the number of hidden layer neurons in the whole training process. Compared with the traditional classification algorithms, ELM has a fast-learning speed and strong generalization capability. These three algorithms have different characteristics and might achieve better prediction results under different conditions or scenarios, so all three algorithms were adopted and compared for the CNC prediction in this study. The number of principal components of the PLS was 6. The training epoch, the learning rate, and the number of hidden layers of the BPNN model were 1000, 0.05, and 22, respectively. The transfer function and the number of hidden layers of the ELM model were sigmoidal function and 50, respectively.

SVM is a classic machine learning method for classification. It maps data from a low-dimensional space to a high-dimensional space through a kernel function and separates the classes with a decision surface that maximizes the margin between the classes. Thus, SVM was selected for the irrigation levels classification in this study. Due to its strong ability to perform nonlinear mapping, BPNN is suitable for not only solving fitting problems, but also classification problems, so BPNN was also selected here for comparison with SVM in the classification of irrigation levels. The penalty factor and the kernel function of the SVM model were 10 and 0.167, respectively. The training epoch, the learning rate, and the number of hidden layers of the BPNN model were 1000, 0.1, and 10, respectively. 

### 2.7. Accuracy Assessment Metrics

*R* and root mean square error (*RMSE*) were used to evaluate the accuracies of the CNC prediction models. *R* was introduced in Section 2.5, and here the correlation between the predicted values and the actual values were calculated to evaluate the accuracies of the prediction models. *RMSE*, which was calculated by Equation (5), can directly reflect the errors of the prediction models.

(5) $$RMSE=\sqrt{\frac{1}{n}\sum_{i=1}^{n}{\left(y_{i}-\hat{y_{i}}\right)}^{2}}$$

where, $y_{i}$ and $\hat{y_{i}}$ represent the estimated value and actual value for sample *i*, respectively.

The confusion matrix is also known as the probability matrix or error matrix [62]. It is a specific matrix for visualizing algorithm performance, and is often used to evaluate the classification results. The rows in the matrix represent the actual irrigation levels and the columns represent the predicted irrigation levels. The confusion matrix is named because it can easily indicate whether multiple classes are confused (that is, one class is expected to be another class). Common indicators including producer’s accuracy (*PA*), user’s accuracy (*UA*), and overall accuracy (*OA*), can be calculated in the confusion matrix. *PA* refers to the ratio of the correctly classified sample numbers in a class to the actual total numbers of that class, also called true positive rate (*TPR*). *UA* refers to the ratio of the correctly classified sample numbers in a class to the classified total numbers of that class, also called positive predictive value (*PPV*). *OA* refers to the ratio of all correctly classified sample numbers to all sample numbers of all the classes. The calculation formulas of the two indicators are shown in Equations (6)–(8).

(6) $$PA/TPR=\frac{TP}{TP+FN}$$

(7) $$UA/PPV=\frac{TP}{TP+FP}$$

(8) $$OA=\frac{TP+TN}{P+N}=\frac{TP+TN}{TP+TN+FP+FN}$$

*TP*, *FP*, *TN* and *FN* represent the numbers of true positive, false positive, true negative, and false negative samples in the classify result, respectively. 

## 3. Results

### 3.1. CNC Prediction

#### 3.1.1. Relation Analysis between the Spectral Features and the CNC

To eliminate the influence caused by different magnitudes of each variable, the datasets were firstly normalized before GRA. In the GRA results listed in Table 4, all the VI had a *GRD* larger than 0.5 with the CNC, while the SRPI had the strongest grey relation of 0.94.

Correlation analysis was also conducted for these ten VIs, and the results were shown in Table 5. Most of the VIs had a very high correlation (*R* > 0.9) with each other, except NGBDI and RVI_2_. Two variables with a higher correlation means more redundant information is contained in them, which means that selecting both as inputs could be avoided. 

Based on the results in Table 4 and Table 5, we can find that though most of the VIs (SRPI, NPCI, RVI, MSRI, NDVI, SIPI, OSAVI and SAVI) had very high *GRD* (>0.8) with the CNC, they also had very high correlations (*R* > 0.9) with each other. Two basic rules, as follows, should be considered in the selection of spectral features, which could help by selecting the more sensitive and less-redundant spectral features as the input for CNC prediction based on the GRA and *R* results: (1) The *GRD* of the selected spectral feature(s) should be relatively high with the CNC (*GRD* > 0.65); (2) The *R* between the spectral feature(s) should be relatively low to avoid introducing redundant information and variable coupling. Therefore, not all the VIs with higher *GRD* could be selected as model inputs. Here, the first two VIs in Table 4, SRPI and NPCI, were recommended as efficient inputs. Furthermore, NGBDI, which had relatively higher grey relational degree with the CNC and a lower *R* with SRPI and NPCI, was also recommended as another efficient input variable.

#### 3.1.2. Modeling with the Five-Band Reflectance

The five-band reflectance of the multispectral image were firstly taken as the input variables, and PLS, BPNN, and ELM algorithms were used to build the CNC prediction models, respectively.

**Figure 6:**
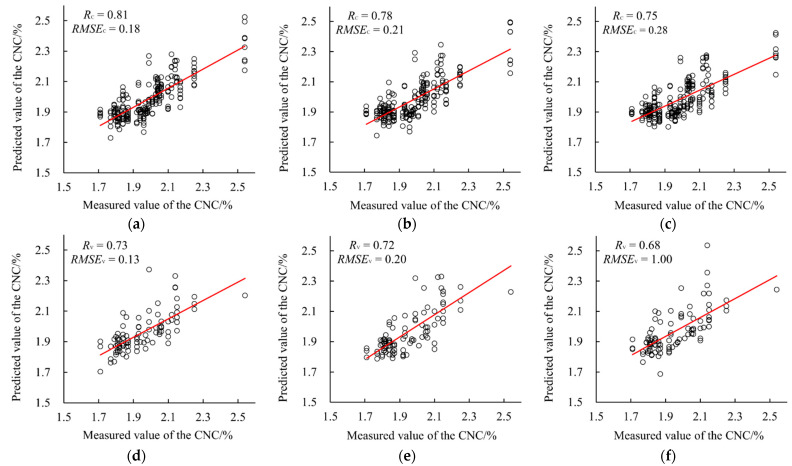
The CNC prediction results of the PLS, and BPNN ELM models based on the five-band reflectance. (**a**) The calibration result of the PLS model; (**b**) the calibration result of the BPNN model; (**c**) the calibration result of the ELM model; (**d**) the validation result of the PLS model; (**e**) the validation result of the BPNN model; (**f**) the validation result of the ELM model.

The modeling results were listed in Table 6, while the scatter plots of the measured values and the predicted values of every model were also presented in Figure 6. As can be seen, the PLS model had the highest *R*_v_ value of 0.73 and the lowest *RMSE*_v_ value of 0.13 in both the calibration set and the validation set compared to the ELM and BPNN model. This indicated that PLS had better modeling performance.

#### 3.1.3. Modeling with VIs

In addition to the three recommended VIs (SRPI, NPCI and NGBDI), SIPI was also selected to build prediction models for comparison. Several models were established from a single VI or a combination of VIs with different modeling algorithms. The results were listed in Table 7. The PLS model still had a more accurate and balanced performance than the BPNN and ELM models. From the results of the single VI-based models, we could find that VIs which had higher GRD with the CNC had better performance in prediction models. From the results of the double and multiple VI-based models, the SRPI- and SIPI-based model showed lower accuracy than the SRPI- and NGBDI- based model, even though SIPI had a higher GRD than NGBDI. This proved the correctness of choosing NGBDI as the supplement variable rather than the others. The model with the highest accuracy (*R*_v_ = 0.63) was established based on SRPI and NPCI and NGBDI, rather than all VIs, indicating that simply increasing the number of input variables does not necessarily improve the accuracy of the model. As long as the variables are selected correctly, fewer variables may bring higher accuracy to the model. 

#### 3.1.4. Modeling with the Five-Band Reflectance and VIs

**Figure 7:**
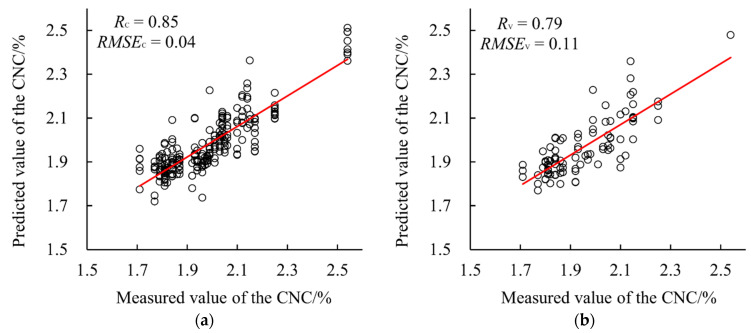
CNC prediction result of the PLS model based on the five-band reflectance combined with NGBDI, SRPI and NPCI. (**a**) The calibration result; (**b**) the validation result.

Other types of models based on different combinations of the five-band reflectance and VIs were established, and the results were listed in Table 8. Of the different modeling algorithms, PLS still had the best performance among the three algorithms, BPNN had slightly lower accuracy than PLS, and ELM had an obvious lower accuracy than the other two algorithms. The PLS model based on FR and the three recommended VIs (SRPI, NPCI and NGBDI) had the highest accuracy, with the highest *R*_v_ of 0.79 and the lowest *RMSE*_v_ of 0.11. The scatter plots of the prediction result of the best model were presented in Figure 7. Higher accuracy based on the five-band reflectance combined with proper VIs was reached because this combination of input variables not only ensured the integrity of information, but also highlighted the spectral characteristic information. The results also demonstrated that the selection of input variables was crucial.

### 3.2. Irrigation Level Recognition

Sugarcane is a crop with high stem, large biomass, and long growth period. It has a large water requirement, as well as being dependent on fertilizers, especially in the early and middle growing stages (the seedling, tillering and elongating stage). Knowledge of water conditions during these stages is of great importance for sugarcane irrigation management.

#### 3.2.1. Correlation Analysis

The correlations between the irrigation amounts and the spectral features, including the band reflectance and ten VIs, were firstly analyzed, as shown in Table 9. As can be seen, regardless of the amount of fertilizer applied, irrigation had high correlations of above 0.65 with red, blue, SRPI, NPCI and NGBDI. Red, blue and NPCI were negatively correlated with irrigation, while SRPI and NGBDI were positively correlated with irrigation. Among the five spectral bands, red, green and blue bands were negatively correlated with irrigation, while red edge and NIR bands were positively correlated with irrigation. This phenomenon is consistent with the spectral variation trend of the green plant (the healthier in growth condition, the lower the reflectance in visible range, and the higher the reflectance in red edge and NIR range).

#### 3.2.2. Classification Results

Based on the five parameters of red, blue, SRPI, NPCI, and NGBDI, which had the highest correlations with irrigations SVM and BPNN were adopted to classify the irrigation levels. The results were listed in Table 10.

As shown in Table 10, the *OA* of SVM for irrigation level recognition was 80.6%, while the *OA* of BPNN was only 61.7%. From the classification results of SVM, the UA and PA of irrigation_0 were the highest, reaching 94.9% and 87.0%, respectively. The accuracies of the other two levels generally exceeded 70%. This result reflects that the larger the difference in irrigation amount, the higher the classification accuracy. In addition, most of the misclassified samples were misclassified due to adjacent irrigation levels. For example, among the 108 samples in Irrigation_0, 94 samples were correctly identified, 13 samples were misidentified as Irrigation_180, and only one sample was misidentified as Irrigation_300. Among the total number of 63 misclassified samples, 62 samples were misclassified into adjacent levels, and only one sample was misclassified into a level far away. This indicated that the identification of irrigation levels using multispectral images had great potential and could be used for the recognition of crop water stress condition.

## 4. Discussion

The results in Table 6, Table 7 and Table 8 indicated that the PLS models had the best performance for sugarcane CNC prediction based on different input combinations, which is consistent with research into citrus CNC prediction by Liu et al. [63], grapevine LNC prediction by Moghimi et al. [64], etc. The sugarcane CNC prediction model showed a highest accuracy of *R* = 0.79 and *RMSE* = 0.11, which was also close to the previous studies by Liu et al. (*R* = 0.65, *RMSE* = 0.13) [63] for the citrus CNC prediction and Moghimi et al. (*R* = 0.74, *RMSE* = 0.23) [64] for grapevine LNC prediction.

Compared to the five-band reflectance models in Table 6, VI-based models in Table 7 had obvious lower accuracy, indicating that taking VIs alone as the input would decrease the prediction accuracy comparing to taking the entire five-band reflectance as the inputs. It reflected that the VIs did not exert their characteristics, which should enhance the spectral features and reduce environmental interference [65]. The main reason was that VIs do have advantages when it comes to reducing the influence caused by uneven light (illumination) and different backgrounds. However, in this study, only one image of a small field was acquired in a very short time, meaning that the light difference and background difference was not significant. This made the contribution of VIs less than that of the whole spectral reflectance [66,67].

Regardless, VIs could still help to improve the modeling accuracy, and this was proved in the results listed in Table 8. Among different combinations of input spectral features, the five-band reflectance combined with the three VIs (SRPI, NPCI, and NGBDI) had the highest accuracy in CNC prediction, with the *R*_v_ of 0.79 and the *RMSE*_v_ of 0.11; this was 8.2% higher in *R*_v_, and 15.4% lower in *RMSE*_v_, than the five-band prediction model (*R*_v_ = 0.73, *RMSE*_v_ = 0.13). 

Moreover, all three of those VIs were calculated from the visible bands (SRPI and NPCI are both calculated from the green and red bands, while NGBDI is calculated from the blue and green bands), indicating that the visible bands contained more sensitive information for sugarcane CNC prediction. Ranjan et al. [68] explored the spectral characteristic of CNC in crops, with characteristic wavelengths mainly in the range of 430 nm, 460 nm, 640 nm, 910 nm, 1510 nm, 1940 nm, 2060 nm, 2180 nm, 2300 nm, and 2350 nm. In this study, the multispectral camera had a spectral range of about 450 nm–850 nm, which contained only the visible sensitive bands. This proves the rationality that the VI selected in this study is mainly concentrated in the visible range. As is generally known, different N inputs could lead to different leaf pigment concentrations, leaf internal structures, and canopy structures [64,69,70]. The visible bands are closely related to leaf pigments and canopy structures and, as such, offer a great potential for N prediction. 

Furthermore, this research also discovered that the irrigation levels could be effectively classified based on the reflectance at red and blue, combined with the SRPI, NPCI and, NGBDI, which were the spectral features in the visible bands. Indeed, the NIR bands are generally more sensitive to plant water content. However, the most sensitive bands which sit between 1480 and 1500 nm are out of the range of the multispectral camera used in this study [69,70]. Insufficient water input could obviously affect plant metabolism, which indirectly affects the leaf pigment concentrations. Therefore, the irrigation level recognition model can achieve better classification accuracy by only using the three visible bands. 

This study has achieved good results for CNC prediction and irrigation level classification based on multispectral remote sensing. Although research on crop monitoring based on UAV multispectral imagery have been widely carried out for more 10 years, but it is rarely applied in wide field management. Several bottlenecks need to be addressed at present. Sozzi et al. [4] compared the advantages and disadvantages of satellite-, plane-, and UAV-based multispectral imagery in variable rate N application in terms of cost, economic benefit, optical quality, and usage scenarios. It was pointed out that although satellite- and plane-based imagery have low optical quality and low resolution, they can provide applicable variable N rate suggestions, and bring economic benefits for large-scaled farms due to their relatively low cost. The UAV platform does have limits in acquisition cost and flight coverage at present. However, as the development of UAV technology and the increase requirement of UAVs, the cost can be significantly reduced and the battery performance can be enhanced in the future. With the emergence of automated UAV base stations and the reduction of image processing costs, the large-scale application of UAVs is just around the corner. By then, UAV remote sensing technology can be widely accepted in farm-scale crop monitoring with its flexible and autonomous acquisition style, high-quality image data, and low-cost validity. 

## 5. Conclusions

High resolution multispectral images of a sugarcane field were collected by UAV, and its ability to predict CNC and irrigation levels was evaluated. The main conclusions were as follows:

1. Ten VIs were used to determine sensitive spectral features for CNC and irrigation level prediction. The SRPI, NPCI, and NGBDI composed of the visible bands were sensitive to the sugarcane CNC as well as the irrigation level, and had a notable contribution to the accuracy improvement of the CNC prediction model.
2. Different modeling algorithms based on different spectral features were compared in predicting sugarcane CNC. The PLS model had a clearly superior performance than the BPNN and ELM models. It was also crucial to select proper features among the band reflectance and VIs. The PLS model, based on the five-band reflectance combined with SRPI, NPCI, and NGBDI, had the highest *R*_v_ of 0.79, and the lowest *RMSE*_v_ of 0.11.
3. Based on the correlation coefficients with irrigation levels, the red band, blue band, SRPI, NPCI, and NGBDI were adopted as the variables to classify irrigation levels based on SVM and BPNN, respectively. SVM reached an obviously superior performance compared to BPNN, with its overall classification accuracy of 80.6%.

High resolution multispectral images have been demonstrated as effective for CNC prediction and water irrigation level recognition. More adequate experiment could be conducted to collect more samples at different growth stages in sugarcane field. Time- or period-dependent prediction models could be studied to give users more accurate information.

## Figures and Tables

**Table 1:** Irrigation amount at different growth stages.

Growth Stage	Irrigation Date	Irrigation Level (m^3^/ha)	
		W0.6	W1.0
Seedling	10 April 2018	60	90
Tillering	29 May 2018	30	60
Elongating	4 July 2018	60	120
Maturing	12 October 2018	30	30
Total		180	300

**Table 2:** The selected VIs and their calculation formulas.

VIs	Calculation Formula	References
Normalized Difference Vegetation Index (NDVI)	(NIR-R)/(NIR + R)	[48]
Modified Simple Ratio Index (MSRI)	$(\mathrm{NIR}/R-1)/\sqrt{\mathrm{NIR}/R+1}$	[49]
Optimized Soil-adjusted Vegetation Index (OSAVI)	1.16(NIR-R)/(NIR + R + 0.16)	[50]
Ratio Vegetation Index (RVI)	NIR/R	[51]
Soil-adjusted Vegetation Index (SAVI)	1.5(NIR-R)/(NIR + R + 0.5)	[52]
Structure Insensitive Pigment Index (SIPI)	(NIR-B)/(NIR + B)	[53]
Simple Ratio Pigment Index (SRPI)	B/R	[54]
Normalized Pigment Chlorophyll Index (NPCI)	(R-B)/(R + B)	[54]
Ratio Vegetation Index 2 (RVI2)	NIR/G	[55]
Normalized Green-Blue Difference Index (NGBDI)	(G-B)/(G + B)	[56]

Note: The spectral reflectance of B, G, R, RE and NIR is at the wavelength of 475 nm, 560 nm, 668 nm, 717 nm and 840 nm, respectively.

**Table 3:** Modeling algorithms.

Models	Validation Method	Algorithms	Ratio
CNC	Hold-out	PLS	7:3
		BPNN	
		ELM	
Irrigation level classification	Three-fold cross validation	SVM	2:1
		BPNN	

Note: Ratio means the calibration set to the validation set.

**Table 4:** GRA results between each VI and the CNC.

VI	*GRD* with the CNC	Rank
SRPI	0.94	1
NPCI	0.93	2
RVI	0.92	3
MSRI	0.89	4
NDVI	0.88	5
SIPI	0.87	6
OSAVI	0.84	7
SAVI	0.82	8
NGBDI	0.70	9
RVI_2_	0.58	10

**Table 5:** Correlations between each VI.

Correlations	SRPI	NPCI	RVI	MSRI	NDVI	SIPI	OSAVI	SAVI	NGBDI	RVI_2_
SRPI	1.00	−0.97	0.98	0.98	0.98	−0.96	0.98	0.97	−0.54	0.15
NPCI		1.00	−0.96	−0.96	−0.96	0.99	−0.98	−0.99	0.60	−0.20
RVI			1.00	1.00	1.00	−0.93	0.99	0.97	−0.51	0.14
MSRI				1.00	1.00	−0.93	0.99	0.98	−0.52	0.14
NDVI					1.00	−0.94	0.99	0.98	−0.52	0.14
SIPI						1.00	−0.96	−0.97	0.62	−0.21
OSAVI							1.00	1.00	−0.53	0.13
SAVI								1.00	−0.53	0.12
NGBDI									1.00	−0.88
RVI_2_										1.00

**Table 6:** CNC prediction results with PLS, BPNN and ELM based on the five-band reflectance.

Input Variables	Algorithm	Calibration Set		Validation Set	
		*R*_c_	*RMSE*_c_	*R*_v_	*RMSE*_v_
Five-band reflectance	PLS	0.81	0.18	0.73	0.13
	BPNN	0.78	0.21	0.72	0.20
	ELM	0.75	0.28	0.68	1.00

Note: *R*_c_ and *RMSE*_c_ represent the *R* and *RMSE* in the calibration set, *R*_v_ and *RMSE*_v_ represent the *R* and *RMSE* in the validation set.

**Table 7:** CNC prediction results with PLS, BPNN and ELM based on VIs.

Input Variables	Algorithm	Calibration Set		Validation Set	
		*R*_c_	*RMSE*_c_	*R*_v_	*RMSE*_v_
SRPI	PLS	0.63	0.13	0.56	0.11
	BPNN	0.80	0.94	0.59	0.89
	ELM	0.73	0.68	0.52	1.59
NPCI	PLS	0.44	0.15	0.58	0.15
	BPNN	0.81	0.62	0.50	1.73
	ELM	0.79	0.86	0.45	1. 95
SIPI	PLS	0.60	0.14	0.54	0.19
	BPNN	0.90	0.26	0.50	0.97
	ELM	0.72	0.67	0.43	0.60
NGBDI	PLS	0.57	0.10	0.42	0.15
	BPNN	0.79	0.89	0.46	1.05
	ELM	0.77	0.92	0.40	1.04
SRPI & NPCI	PLS	0.60	0.15	0.57	0.15
	BPNN	0.83	0.21	0.58	1.14
	ELM	0.80	0.86	0.49	0.98
SRPI & SIPI	PLS	0.49	0.15	0.49	0.17
	BPNN	0.72	1.28	0.48	1.25
	ELM	0.81	0.60	0.44	1.04
SRPI & NGBDI	PLS	0.53	0.12	0.55	0.18
	BPNN	0.76	0.96	0.54	1.30
	ELM	0.71	0.86	0.51	1.27
SRPI & NPCI & NGBDI	PLS	0.64	0.14	0.63	0.14
	BPNN	0.74	1.17	0.62	1.26
	ELM	0.75	1.73	0.62	1.96
Ten VIs	PLS	0.65	0.12	0.52	0.16
	BPNN	0.81	1.51	0.52	1.12
	ELM	0.78	1.62	0.50	1.19

Note: *R*_c_ and *RMSE*_c_ represent the *R* and *RMSE* in the calibration set, *R*_v_ and *RMSE*_v_ represent the *R* and *RMSE* in the validation set.

**Table 8:** CNC prediction results with PLS, BPNN and ELM based on different input variables (FR represents five-band reflectance).

Input Variables	Algorithm	Calibration Set		Validation Set	
		*R*_c_	*RMSE*_c_	*R*_v_	*RMSE*_v_
FR & SRPI	PLS	0.82	0.08	0.71	0.17
	BPNN	0.91	0.01	0.72	0.14
	ELM	0.90	0.02	0.64	1.07
FR & SRPI & NPCI	PLS	0.82	0.14	0.72	0.27
	BPNN	0.92	0.01	0.66	0.39
	ELM	0.84	0.01	0.60	0.69
FR & SRPI & NPCI & NGBDI	PLS	0.85	0.04	0.79	0.11
	BPNN	0.87	0.13	0.79	0.39
	ELM	0.84	0.24	0.68	1.31
FR & ten-VIs	PLS	0.81	0.19	0.72	0.68
	BPNN	0.93	0.01	0.69	1.26
	ELM	0.84	0.18	0.53	1.68

Note: *R*_c_ and *RMSE*_c_ represent the *R* and *RMSE* in the calibration set, *R*_v_ and *RMSE*_v_ represent the *R* and *RMSE* in the validation set.

**Table 9:** Correlation analysis result between the spectral features and the irrigation levels.

Spectral Features		*R* between the Spectral Features and the Irrigation Levels
Spectral reflectance	NIR	0.48
	Red edge	0.25
	Red	−0.71
	Green	−0.45
	Blue	−0.69
VI	NDVI	0.21
	MSRI	0.25
	OSAVI	0.29
	RVI	0.28
	SAVI	0.34
	SIPI	0.18
	SRPI	0.65
	NPCI	−0.68
	RVI_2_	0.33
	NGBDI	0.75

**Table 10:** Confusion matrix of the classification results of the irrigation levels.

Classifier		SVM				BPNN			
	Predicted Class	Irrigation_0	Irrigation_180	Irrigation_300	PA	Irrigation_0	Irrigation_180	Irrigation_300	PA
Actual Class									
Irrigation_0 (108 samples)		94	13	1	87.0%	80	25	3	74.1%
Irrigation_180 (108 samples)		5	89	14	82.4%	15	61	32	56.5%
Irrigation_300 (108 samples)		0	30	78	72.2%	2	47	59	54.6%
Total		99	132	93		97	133	94	
UA		94.9%	67.4%	83.9%	*OA* = 80.6%	82.5%	45.9%	62.8%	*OA* = 61.7%

## Data Availability

The data presented in this study are available on request from the author (X.L.).
